# Ion Microprobe Study of the Polarization Quenching Techniques in Single Crystal Diamond Radiation Detectors

**DOI:** 10.3390/ma15010388

**Published:** 2022-01-05

**Authors:** Mauricio R. Ramos, Andreo Crnjac, Donny Cosic, Milko Jakšić

**Affiliations:** Laboratory for Ion Beam Interactions, Ruđer Bošković Institute, Bijenicka Cesta 54, 10000 Zagreb, Croatia; Andreo.Crnjac@irb.hr (A.C.); Donny.Domagoj.Cosic@irb.hr (D.C.); Milko.Jaksic@irb.hr (M.J.)

**Keywords:** sc-CVD diamond, polarization, IBIC, microprobe, quenching techniques

## Abstract

Synthetic single crystal diamond grown using the chemical vapor deposition technique constitutes an extraordinary candidate material for monitoring radiation in extreme environments. However, under certain conditions, a progressive creation of space charge regions within the crystal can lead to the deterioration of charge collection efficiency. This phenomenon is called polarization and represents one of the major drawbacks associated with using this type of device. In this study, we explore different techniques to mitigate the degradation of signal due to polarization. For this purpose, two different diamond detectors are characterized by the ion beam-induced charge technique using a nuclear microprobe, which utilizes MeV energy ions of different penetration depths to probe charge transport in the detectors. The effect of polarization is analyzed by turning off the bias applied to the detector during continuous or discontinuous irradiation, and also by alternating bias polarity. In addition, the beneficial influence of temperature for reducing the effect of polarization is also observed. Finally, the effect of illuminating the detector with light is also measured. Our experimental results indicate that heating a detector or turning off the bias, and then applying it during continuous irradiation can be used as satisfactory methods for recovering the CCE value close to that of a prepolarized state. In damaged regions, illumination with white light can be used as a standard method to suppress the strength of polarization induced by holes.

## 1. Introduction

Semiconductor particle detectors based on silicon are essential in most modern high energy physics experiments, synchrotron imaging applications, etc. [1,2], however, new frontiers in physics, such as those found in nuclear reactors or in outer space, will exceed the limits or even disable some radiation diagnostics [3]. Finding an alternative to current detection systems is crucial for use in the working conditions of new hostile environments with harsh radiation and high temperature [4]. As an alternative to silicon, natural diamonds have been considered to be an optimal material for improving the performance of radiation monitors in such types of environments, but, for years, their cost and availability have limited their use as radiation detectors [5]. Synthetic single crystal diamonds grown using the chemical vapor deposition (CVD) technique [6] constitute excellent candidates for such operations due to their unique electronic properties, i.e., larger thermal conductivity, resistivity, and band gap (≈5.5 eV), which assures detectors with low leakage current even in high-temperature environments. The atom displacement energy (≈43 eV) allows long-term operations in harsh environments, presenting an excellent resistance to radiation [7].

The charge transport properties, with larger mobilities of the free carriers, result in faster responses [8] to ionizing radiation. All these properties, together with the quality, i.e., a good intrinsic diamond material has an extremely low amount of carriers in the conduction band and lower cost, have led to the use of CVD diamonds for electronic applications. Nowadays, diamonds are being used as tracking detectors at the Large Hadron Collider (LHC) of CERN [9], dosimeters in medical applications for radiation therapy [10,11], etc. The capability of using this type of detector as a fast-ion loss detector in ITER [12] and as beam monitoring sensors at the SuperKEKB collider are currently being investigated [13].

**Figure 1:**
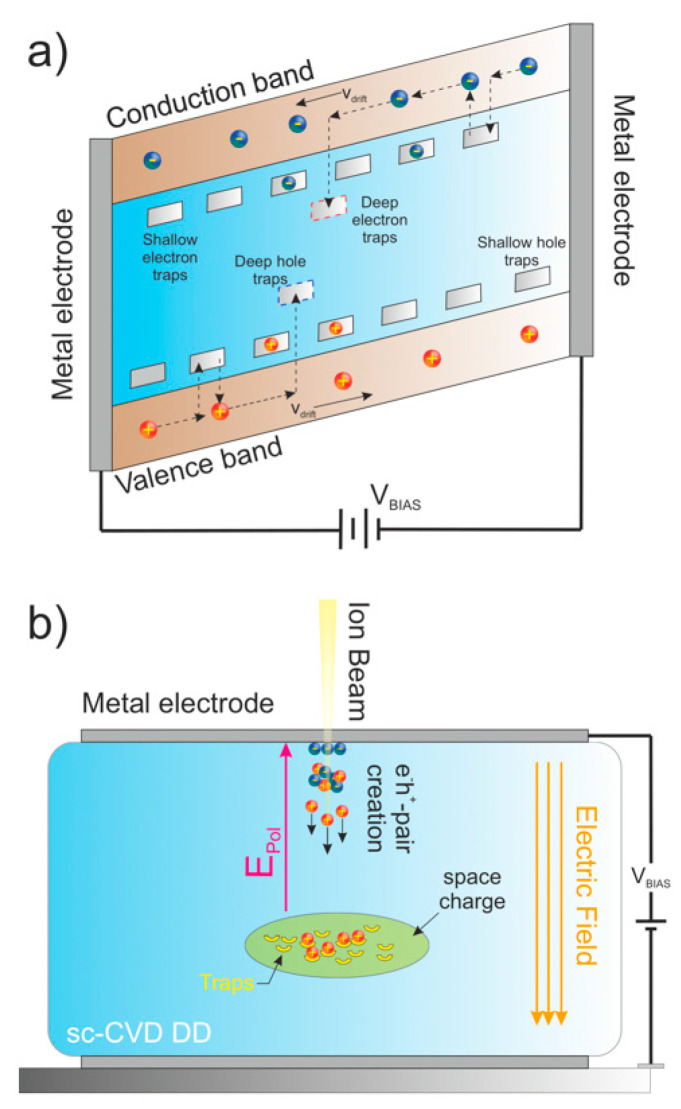
(**a**) Energy band diagram showing the drift motion of the free carriers and trapping/de-trapping process under the presence of an electric field; (**b**) diagram of the polarization phenomena.

The feasibility of using sc-CVDs as particle detectors relies on the principle of signal induction on metal electrodes by moving charges [14]. When a charged particle (electrons, ions, etc.) or incoming radiation (neutrons, x-rays, γ-rays, etc.) enters the sensitive volume of a diamond sample, the radiation deposits energy and ionizes the medium as it passes, generating a certain number of electron-hole pairs, known as free carriers. For diamond, the average energy to produce one electron-hole pair is 13.6 eV. These electrons and holes start to move under the presence of the applied electric field, until they are collected by the electrodes. This motion of charge carriers induces a measurable current at the readout electrode according to the Shockley–Ramo theorem [15,16] and this current can be processed with appropriate pulse processing electronics to obtain the pulse height spectrum of the radiation, therefore, allowing the spectroscopic characterization of the incoming radiation. In an ideal detector, all the charge is collected, and the pulse height spectra appear as the full energy peak. However, in wide band gap materials [17], such as single-crystal diamonds, the free carriers can be captured (Figure 1a) during their motion by electrically active traps, such as intrinsic defects in the lattice or defects induced by radiation [18]. The filling of the traps and the trapping/de-trapping rates of many charge carriers produce an asymmetrical space charge distribution in the sensitive volume of a diamond crystal [19]. The net effect of this asymmetry is the generation of one internal electric field, which is opposite to the electric field induced by the applied bias to the detector.

A reduction in the electric field increases the probability of free carrier recombination, which can produce progressive deterioration of the charge collection efficiency (CCE) from the moment the detector is exposed to radiation. This phenomenon is known as polarization (Figure 1b) and constitutes a serious disadvantage for natural and synthetic diamonds used in many experimental applications [20,21].

Some authors have demonstrated that the ion beam-induced polarization in high resistivity materials such as CdZnTe and CdTe is a complex process dependent on trap density, re-emmision properties (de-trapping) of the free carrier from the trap levels, applied voltage, deposited energy, etc. [22]. In addition, some authors have reported a more powerful polarization with the level of damage accumulated in the detector. Regarding polarization, two effects must be considered: (1) bulk polarization, trapping of the charge in the traps in the bulk of material and (2) surface polarization trapping of the charge on the interface between the material and metallic contact. Surface polarization strongly depends on the type of the contact, connection, etc. Usually, it is visible when the beam is traversing through the whole detector, and therefore bulk polarization is almost negligible in such a setup. Bulk polarization occurs in deep traps of the material (around the middle of the band gap) where the charge cannot release thermally.

The literature exploring the polarization phenomena in diamond is scarce and mainly explores the deterioration of spectroscopy and charge transport properties using the ion beam-induced charge (IBIC) technique and the transient current technique (TCT) performed with radiation emissions such as alpha and beta sources [23]. These studies have all reported a progressive deterioration in the spectroscopic properties of the analyzed devices induced by the polarization, however, the experimental conditions and the physics underlying the polarization mechanisms are not well understood. The IBIC technique is an analytical tool based on the creation of electrons/holes by ionizing radiation using single ions [24]. The IBIC technique using a nuclear microprobe relay to control the type of ionizing radiation, allows for the selection of the ion species and the energy, the use of focused ion beam with micrometer dimensions, and the possibility of raster scan selected areas with excellent lateral resolution. The IBIC is the most frequently used technique for studying spectroscopy and transport properties in semiconductor detectors. In our previous work, we proved that the IBIC technique [25,26] is an excellent tool for imaging and monitoring the influence of polarization on detector performance. In particular, we showed that short-range ion microbeams could be used very successfully to induce instantaneous polarization even in pristine regions [27]. This is due to the fact that bulk polarization occurs in the regions of the detector where only one carrier exists. Therefore, short ion probes are an ideal tool for polarizing material.

All the physical mechanisms that can break or alter this asymmetry are good candidates as reliable methods to depolarize a detector and to recover the nominal electric field. In normal conditions, the natural release of this charge can happen in hours or even days, however, in the literature, different techniques of releasing the charge from such traps have been proposed for wide band gap semiconductors.

The strength and the polarity of the applied electric field have been reported as key parameters associated with the behavior of the charge carrier transport during polarization. Holmes et al. [28] proposed a method to depolarize a metal-insulator-metal (MIM)-structured detector which used periodic forward bias pulses, where recombination took place by allowing the charge of opposite polarity to neutralize the trapped charge. As soon as both carriers were in volume, there was a high probability that the opposite charge would be attracted to the trapped charge and recombine with it. The influence of turning off the bias during irradiation with the same probe that caused polarization has been proposed as one of the easiest ways to depolarize the material [29].

Manfredotti et al. [30] demonstrated that exposing detectors to light (optical excitation) could recover the signal to its initial state, and showed that light had some influence on trap levels and charge carriers. Different studies have exhibited the effect of increasing the temperature on the performance of diamond detectors, however, the available experimental results are contradictory. Tanimura et al. [31] observed a remarkable decrease in signal output with an increase in detector temperature related to polarization. Conversely, other authors [32] have reported a decrease in polarization strength when a detector was heated due to thermal excitation, which increased the thermal de-trapping of carriers from traps filled with radiation, and therefore reduced the accumulated space charge. Andreo et al. [33] did not observe any effect on polarization due to temperature.

A complete description exploring all these techniques applied to a diamond sensor has not been done in detail. In this study, we address the development of approaches to suppress or attenuate this phenomenon. In this study, the IBIC technique was used to study the properties of the polarization phenomena under the influence of these quenching techniques in different sc-CVD diamond detectors. Different settings of the ion beam species (H, C, and He), energies (tuned to explore from shallow to intermediate-range beams), pulsed bias, operating temperature, and illumination conditions were studied. To perform these measurements, a new upgrade of the Ruđer Bošković Institute (RBI) [34] microprobe (including a new heating system, a high voltage (HV) alternating unit, and a light source) was carried out.

This paper is organized as follows: In the Introduction, we explain the drawback of the polarization phenomena when using diamond crystal as a radiation detector; in Section 2, we describe methods to suppress this phenomenon, the detectors, and the experimental setup; in Section 3, we present the main experimental results and a brief interpretation; and finally, in Conclusions, we present the main conclusions related to determining the most efficient method to depolarize.

## 2. Materials and Methods

Measurements were performed on a set of two detectors of different thicknesses to study the proposed quenching techniques on the behavior of the polarization phenomena. Electronic grade single-crystal CVD diamonds used for the construction of the detectors were provided by Element Six (Didcot, UK) [35]. The crystals have dimensions of 3 × 3 mm^2^ with a thickness of 65 μm (detector A) and 1.5 × 1.5 mm^2^ with a thickness of 500 μm (detector B). The nominal impurity concentration of both crystals is below 5 ppb for nitrogen and 1 ppb for boron. The front and back surfaces of the crystal were coated, using the sputtering evaporation technique with tungsten electrodes of 200 nm thickness [36]. The coated samples were mounted on specially designed ceramic printed circuit boards (PCBs) with gold contacts, which could be used even at high temperatures.

The experiments to irradiate the detectors were performed using the ion beam microprobe line [37] at the RBI in Zagreb using ions from the 6 MV tandem Van der Graaff accelerator (High Voltage Engineering Corporation, Wakefield, MA, USA) [38]. The CCE was measured with protons, helium, and carbon beams focused to a micrometer size and with beam currents of ≈1 kcps. Focused ions are particularly good for studying polarization since it is possible to tune the ion beam energy (fixed range) which allows one to probe the charge transport properties of detectors from shallow to intermediate depths, with excellent spatial resolution [39]. For the performance of the IBIC measurements, the detectors were mounted inside the vacuum chamber, perpendicular to the ion beam, and were irradiated through the front biased electrode, while the backside electrode was grounded. The IBIC pulses were processed using the standard electronic chain for nuclear spectroscopy [40,41].

Lower electric fields (E < 0.5 V/μm) were used to enhance polarization due to the fact that, at higher electric fields, the charge de-trapping rate increases, and thus reduces polarization induced problems [27]. For thermal excitation, a new ceramic heater [42] with a temperature control system was used. Despite the fact that the high thermal conductivity in sc-CVD crystals [43] assures a quick thermal equilibrium between the heater and the diamond, all the measurements were performed 5 min after the thermocouple stabilized to the operation temperature (±5 °C).

For the experiments where the applied bias was switched on/off, a standard detector bias supply source was manually switched during the irradiation. Due to the internal capacitance and resistance of the diamond detector, the electric field is not re-established immediately after the bias is turned on, and also some time is needed for the voltage to reach a zero value when the source is switched off. For this reason, the suitable durations of these cycles were selected to be longer than the characteristic times related to the used biasing electronics. For the alternating bias experiment, an HV device was constructed in-house to switch the bias during detector operation. This device was based on the fast high-voltage push-pull switch system supplied by Behlke [44]. Two DC power supplies (one with positive voltage and the other one with negative voltage) were connected to the device and the output signal was applied to the detector as a periodic rectangular voltage pulse between the two biases applied. The system could control and vary the lengths of the pulses for each polarity and the duty cycles were controlled by a graphical interface managed by the acquisition software Spector [45].

**Figure 2:**
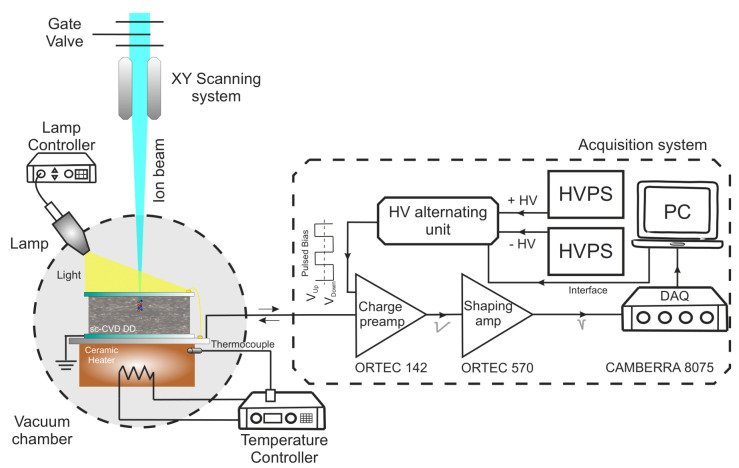
Schematic diagram showing the main components of the experimental setup and the standard spectroscopy acquisition chain.

Finally, for testing the influence of white light on the charge transport during polarization, a standard light source to illuminate the sample during the irradiation was installed in one dedicated port of the vacuum chamber. The spectral emission of the light source was measured to have a maximum emission centered around 592 nm. For the I-V acquisition, a Keithley Picoammeter with a programmable voltage source [46] was used. A schematic diagram of the experimental setup outlining the new upgrades and the spectroscopy acquisition chain is shown in Figure 2.

Experiments at different temperatures were performed by the IBIC characterization and mapping of the virgin and previously irradiated areas of the 500 μm diamond detector.

Short penetrating beams of 3 MeV He^++^ ions with a range of ≈5.8 μm in diamond were used. During these sets of experiments, the bias applied to the detector was ±225 V (E = ±0.45 V/μm). Before irradiation, the detector was heated to each of the selected temperatures: 24 °C (room temperature), 95 °C, and 175 °C.

For switching the bias on/off and the alternating bias experiments, the 65 μm detector was tested by means of a 4 MeV C^3+^ microbeam. Since the range of these ions is only ≈2.0 μm, they can also be considered to be shallow probing ions. During these experiments, the bias was set to ±12 V (E = ±0.18 V/μm) and several different time periods, where the bias was applied or interrupted, were carried out. The IBIC tests presented in this study were done in 30 s duration of bias on/off cycles, while ion beam irradiation was kept either continuous or discontinuous (off when bias is off). In the case of alternating bias experiments, two different duty cycles were used, as listed in Table 1.

The use of shallow probing beams allows one to study the charge transport properties induced by only one type of free carrier [47]. In our experimental configuration, the induced current was mainly governed by the holes when a positive polarity was applied, while negative polarity induced currents that were governed mainly by electrons motion. In our previous work, we observed that a strong polarization effect was associated with radiation-induced damage. Furthermore, in damaged regions, we observed a certain influence of light in the evolution of the CCE when polarization was present.

**Figure 3:**
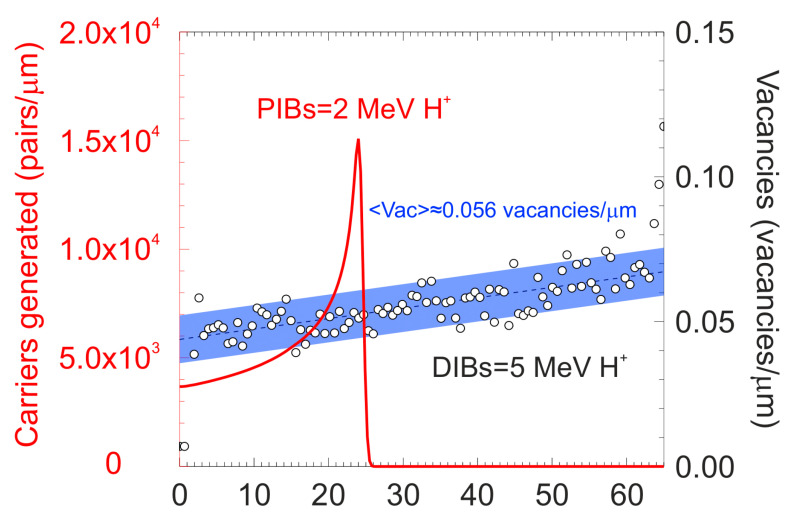
Ionization profile (red curve) for a 2 MeV H^+^ beam impinging in diamond. The vertical units are the number of electron/hole pairs generated per unit length. (Right) Vacancy profile (white points) for a 5 MeV H^+^ ion beam in the diamond.

To explore the influence of optical excitation on polarization in more detail, the 65 μm diamond detector was damaged by means of a traversing 5 MeV proton microbeam. For this purpose, 3 areas (scanned area of ≈100 × 100 μm^2^) were irradiated on the microprobe line at different fluences ranging from 8.6 × 10^12^ to 1.7 × 10^14^ ions/cm^2^. Subsequently, the IBIC probing of the entire sample was carried out using a 2 MeV H^+^ beam (intermediate range). The generated charge distribution of the probing ion beam (PIBs) and the vacancy profile of the damaging ion beam (DIBs) were obtained with SRIM [48]. The vacancy profile of the damaging ion beam showed a homogeneous production of points defects, with an average of 0.056 vacancies/μm × ion, throughout the whole diamond thickness, where both charge carriers could be trapped. The ionization profile was represented as the total number of free carriers generated per unit length, considering that an average energy creates one e/h pair in diamond of 13.6 eV. The profiles are both displayed in Figure 3.

**Figure 4:**
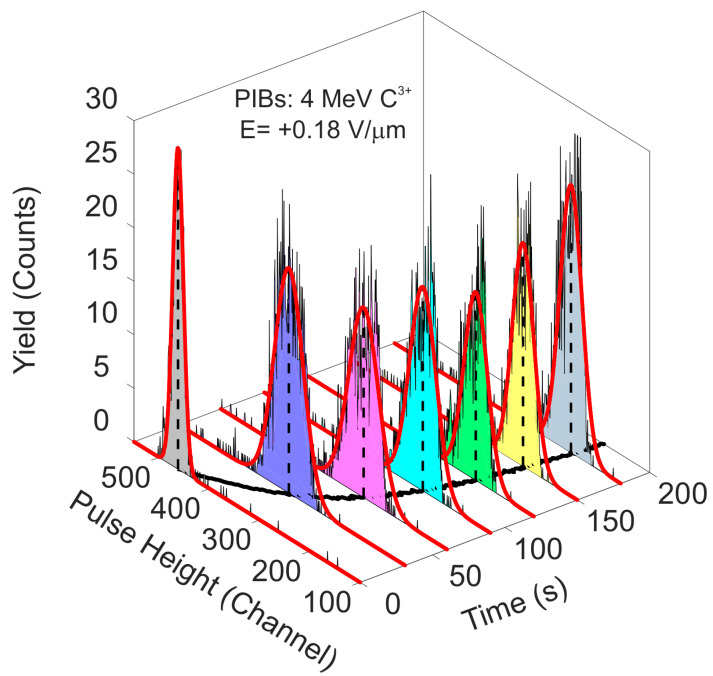
Train of histograms at different times from the beginning of the irradiation obtained with IBIC using a 4 MeV C^3+^ microbeam.

During the performance of all IBIC measurements, the acquisition system recorded the coordinates (x,y) where the events took place and the associated channel (energy), and the time of the event occurrence. All this information was saved in the memory of the data acquisition system and transferred to a raw file for a post-processing analysis. The quantification of the temporal evolution of the pulse height spectrum was carried out with a Matlab code that extracted from the raw file all the events that had taken place in a fixed interval of time. The full spectra were decomposed in a train of consecutive histograms in order to obtain the continuous degradation of the signal during polarization. These sequences of spectra were analyzed to obtain the most probable centroids from a Gaussian fit and the CCE values were calculated from the centroids of the peaks. A tail pulse generator and an Si detector mounted in the vacuum chamber were used for the calibration of the processing electronic chain, which allowed for the conversion of the pulse height channel in the CCE (assuming that the Si detector has 100% of the CCE for the same irradiation configuration). An example of the described data analysis processing for the 65 μm detector is shown in Figure 4.

In this example, a 4 MeV C^3+^ microbeam uniformly scanned one selected area (≈75 × 75 μm^2^) of the pristine region of the detector. The IBIC was monitored during irradiation to record the events over the time period using the acquisition system. In the offline analysis, the pulse height histograms were constructed setting the exposure time to one second. In the previous figure, the pulse height spectra at different times were plotted together with the best Gaussian fit overlapping the spectra.

As can be seen in Figure 4 above, the pulse height histograms continuously shift to lower channels as irradiation progresses, which is indicative of a continuous decrease in the CCE value due to polarization. In the XY plane, the positions of the centroids of the pulse height histograms were plotted (in black) as a function of time. This was the figure of merit for studying the temporal evolution of CCE [49].

## 3. Results and Discussion

### 3.1. Leakage Current

**Figure 5:**
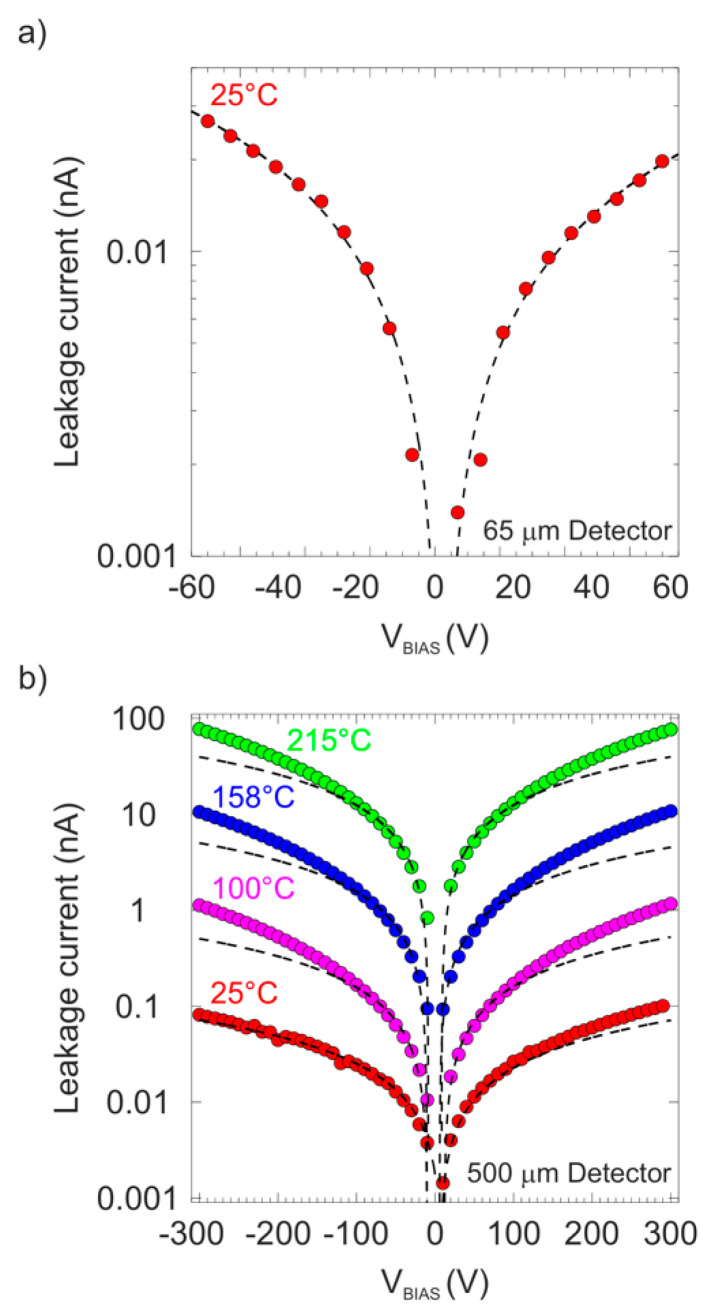
(**a**) Leakage current vs. applied bias for the 65 μm detector at room temperature; (**b**) Leakage current vs. applied bias for the 500 μm detector at four different temperatures.

Figure 5a shows the measured detector leakage current versus the applied bias voltages (ranging from −70 to 70 V) for the 65 μm diamond detector.

This detector was used only in applications without heating, so only one measurement at room temperature was performed. As can be seen, the leakage current presents a linear behavior (dashed black lines correspond to the best linear fit for each polarity) with the applied biases for both polarities, which is consistent with an ohmic device, keeping the leakage below 30 pA for biases around ±65 V (E = ±1 V/μm).

A 500 μm diamond detector was used to study the effect of temperature on polarization phenomena. In Figure 5b, the measured leakage current as a function of the applied biases for the 500 μm detector is shown at different temperatures from room temperature (RT) to 215 °C. This temperature range covered that used during the IBIC characterization and the selected bias scanning was adapted to its particular thickness. Similar ohmic behavior was obtained for the measurement at room temperature. However, with increasing temperature, linearity was only presented for bias below 100 V.

The dashed black lines correspond to the best linear fit for biases below 100 V for both polarities, showing the deviation between the experimental data and the linear relation around this voltage. In addition, an increase in the leakage value was observed with an increase in the temperature, changing the leakage from 10 pA at room temperature to around 100 nA at temperatures around 215 °C. For temperatures above 220 °C, instabilities appeared in the detector and an exponential increase in the leakage was observed, changing the leakage current more than one order of magnitude under small increases in the operating temperature. For this reason, the IBIC measurements were limited to temperatures below 200 °C and 225 V bias for a satisfactory performance of the detector. Note that, due to the wide range of values of the measured leakage between room temperature and 215 °C, in both graphs, the y-axes are represented in the logarithmic scale.

### 3.2. Thermal Excitation

**Figure 6:**
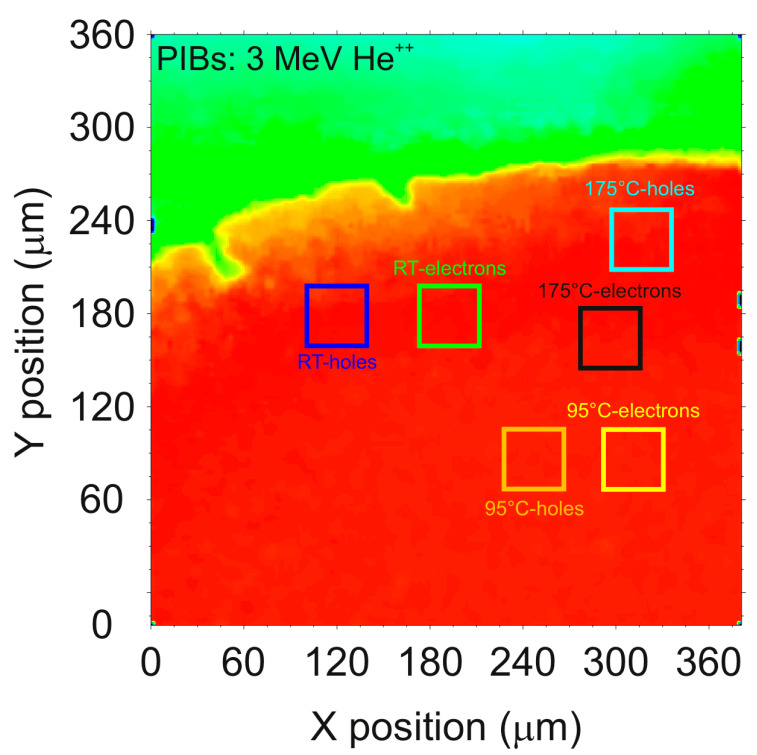
IBIC map of the 500 μm diamond detector biased at +225 V under irradiation with 3 MeV He^++^. The color squares in the pristine region delimit the selected regions used for the characterization where the operating temperature and polarity were set to a fixed value.

To study the influence of polarization quenching using thermal excitation, the temporal evolution of CCE was measured for three different temperatures and for both charge carriers. For this purpose, 6 regions with the same dimensions were defined at different positions of one unirradiated area of the detector, as illustrated in the IBIC map shown in Figure 6.

**Figure 7:**
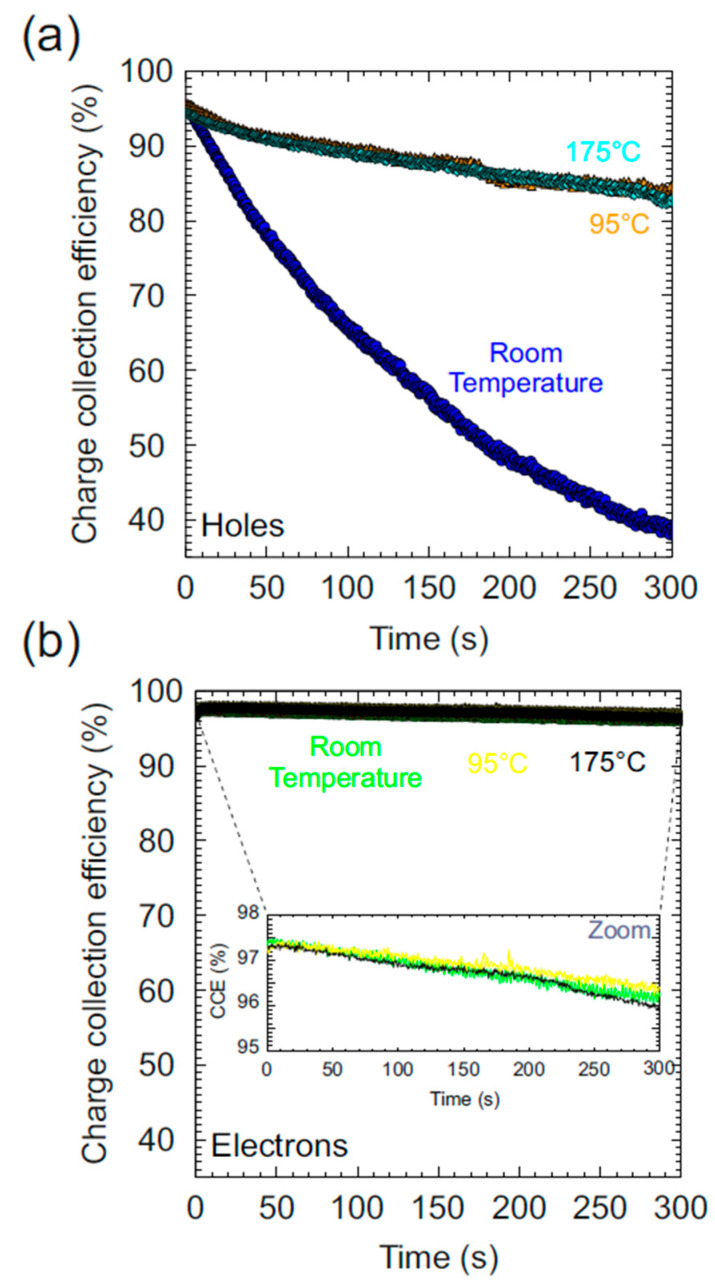
Temporal evolution of CCE at three different temperatures: (**a**) for holes; (**b**) for electrons.

In the map above, the scanned area was close to the edge of the detector, therefore, the homogeneous pristine region (shown in red) and the region outside the detector (shown in green) were clearly distinguishable. For the measurement of each individual region, the temperature and the polarity (positive for holes and negative for electrons) were set to the values shown in the previous map. The detector was operated with the bias voltage kept at ±225 V (E = ±0.45 V/μm), close to the limit where the leakage current deviates from linearity. During IBIC characterization, the heated regions of interest (ROIs) were exposed to an ion beam of 3 MeV He^++^ focused to a micrometer size to scan over the selected area at a count rate of above 1 kcps during 300 s. Because of the limits imposed by the leakage current, the working temperature in this study never exceeded 200 °C. During the processing analysis, the temporal evolution of CCE was obtained setting the acquisition time to 0.5 s per histogram. Figure 7 shows a comparison of the temporal evolution of CCE for three different temperatures from RT to around 200 °C for holes (Figure 7a) and for electrons (Figure 7b).

In Figure 7, the instantaneous degradation of CCE for holes at RT due to polarization can be observed. During the irradiation at RT the CCE suffers a decrease close to 60% of its initial value after 5 min. With increasing temperature, the decrease in CCE remains, but is much less apparent with respect to RT. It is noticeable that the shape of the temporal evolution of CCE is practically the same for 100 °C and 175 °C, showing no significant changes. Under these conditions of heating, the percentage of decrease in the value of CCE was reduced to ≈13% of the initial value in the same interval of time. In fact, for the case of polarization induced by electrons, a weak drop of ≈2% in the CCE was observed for the three different temperatures (clearly visible in the central zoom panel shown in Figure 7b). Therefore, no polarization (or soft polarization) appears in this detector from RT to 200 °C for electrons as described above. 

The presence of strong polarization for holes and not significant polarization for electrons has been reported in several studies and has been related to the density of traps for each type of free carrier and the quality of the sc-CVD [27].

The partial restoration of CCE for holes (between RT and 100 °C) is related to an increase in the probability of de-trapping rate due to thermal re-emission [50], which allows the liberation of the trapped free carriers partially reducing the accumulated space charge.

### 3.3. Bias On/Off

**Figure 8:**
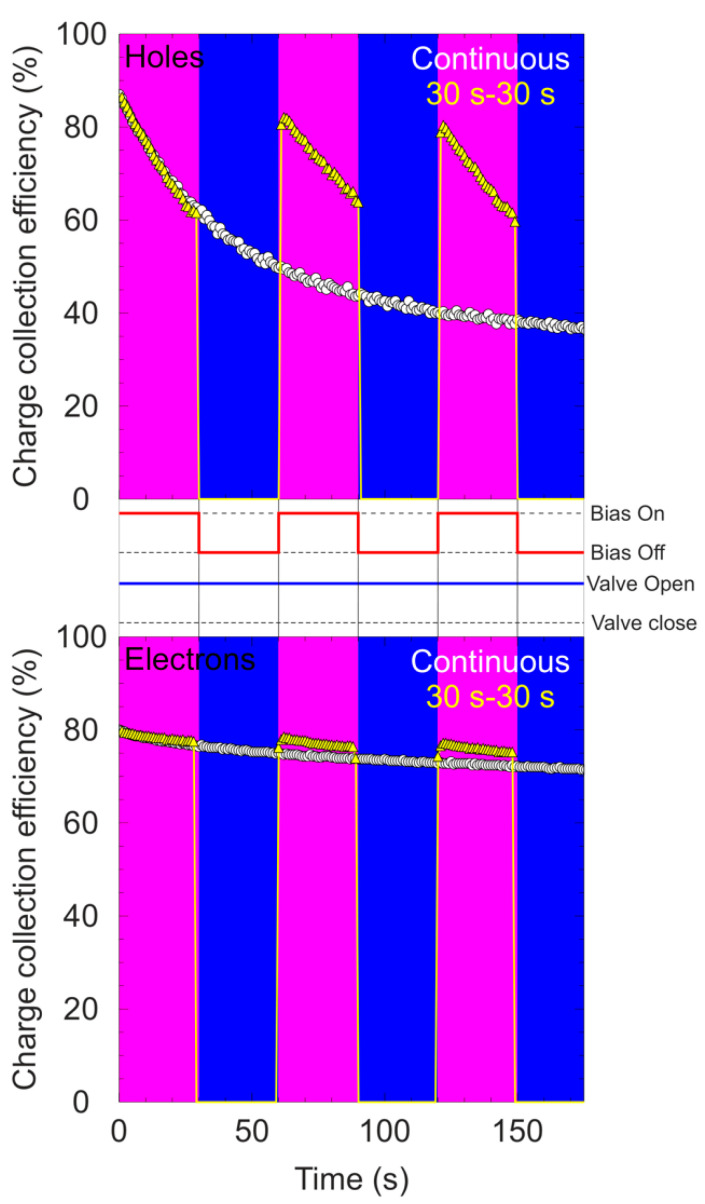
Temporal evolution of CCE (yellow points) for holes (**top**) and for electrons (**bottom**) switching the bias on/off during continuous irradiation. The white points correspond to the cases where the beam and the bias were applied all the time.

The effect on polarization by switching on/off the bias during continuous irradiation was also explored. Following the same protocol explained in the previous paragraphs, different regions were defined in a pristine area of the detector. The temporal evolution of CCE was obtained with the IBIC technique exposing the selected areas to a 4 MeV C^3+^ beam during 180 s at a count rate of around 1 kcps. During these measurements, the bias was on during 30 s and was manually turned off for another period of 30 s. This duty cycle (DC = 50%) was repeated at least three times. The ion beam was always on in the sample during these measurements. All these evaluations were carried out with the 65 μm diamond detector in ambient conditions (room temperature, no illumination), and the behavior of both charge carriers was investigated. The main results and the clock diagram of the applied biases are depicted in Figure 8.

In order to compare these measured values, the temporal evolution of CCE under irradiation with continuous bias and beam is also presented.

The pulse height spectra were recorded setting the acquisition time to 1 s per histogram. For the measurement where the bias and the beam were applied simultaneously (continuous case), a strong decrease in the value of CCE can be observed for holes, dropping the efficiency by more than 50% after 180 s of continuous irradiation. The behavior is different for electrons where the decrease in the value of CCE is slower, reaching a maximum drop of 10% during the same irradiation time. For the measurement where the bias is switched on/off, the behavior is significantly different. For the polarization induced by holes, during the first cycle of 30 s, the same evolution of CCE was observed. 

**Figure 9:**
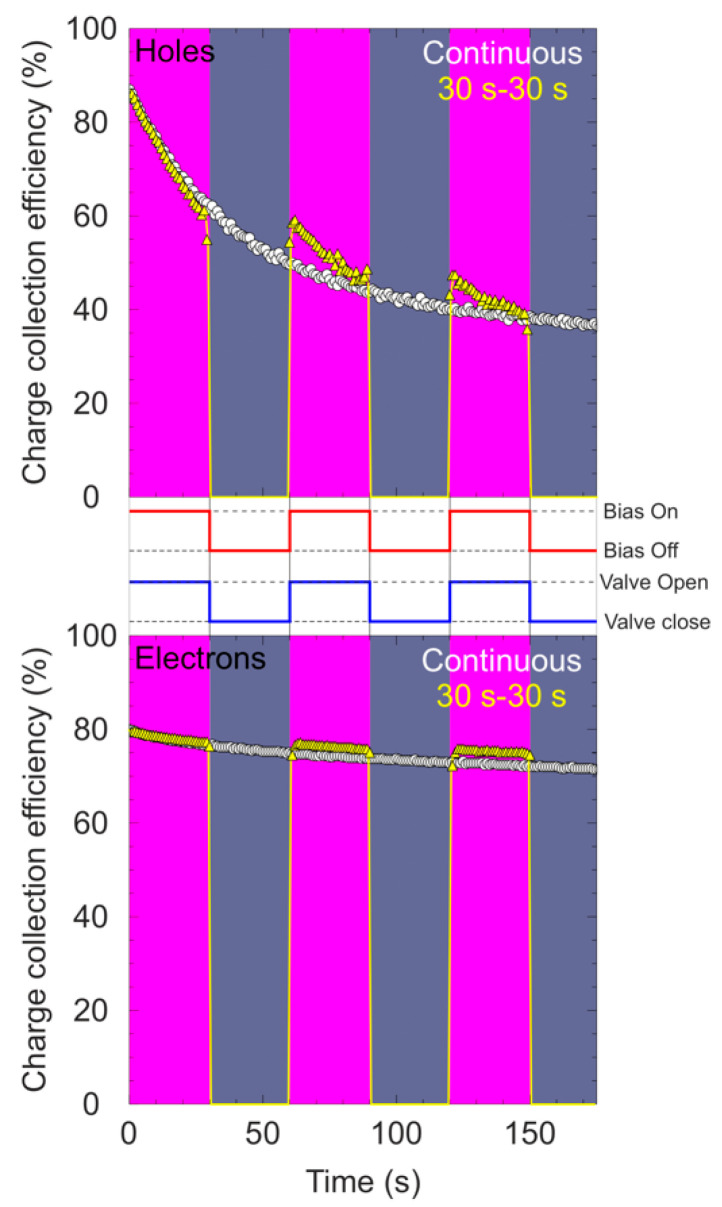
Temporal evolution of CCE (yellow points) for holes (**top**) and for electrons (**bottom**), while switching the bias and the beam on/off. The white points correspond to the case where the beam and the bias were on all the time. The detector was biased at ±12 V.

This behavior was expected because, until this moment, no changes with respect to the continuous case were applied. After 30 s of not biasing the detector, the bias was reapplied. A clear recovery of the CCE value with respect to the value reached before switching off the bias was obtained, however, the decrease in CCE value rapidly continued. This recovery was observed in the three different cycles applied to the detector during this measurement. For electrons, a slight recovery was observed, but due to the fact that the polarization induced by electrons was soft, the recovery was not as intense as the recovery observed for holes, or at least the effect remained negligible. The influence on the stability of the detector while closing the ion beam during the time intervals where the detector was unbiased was also studied. During these measurements, the bias and the beam were on for 30 s and the bias was manually turned off for a period of 30 s. During this period, the ion beam was stopped by closing a gate valve. Figure 9 presents the results for the temporal evolution of CCE for electrons and holes using a 50% duty cycle. In addition, a complete description of the temporal intervals at which the bias voltages were applied can be found in the clock diagram located between the graphs in Figure 9. Again, for polarization induced by hole, the typical deterioration of the output signals was measured during the time that the beam and the bias were on.

After 30 s of removing the bias and the beam, they were established again. It can be seen that contrary to the recovery showed with the continuous beam, no noticeable restoration of the signal was obtained by applying the bias and the beam again. As shown in Figure 9, it can be seen that the signal starts from the same position as the one reached before the bias/beam were switched off and rapidly continues to decrease, and therefore the effect seems to be less pronounced. The same recurring behavior can be observed during the three cycles.

For electrons, the results are quite similar to the case with a continuous beam showing no significance recovery. In addition, the differences with respect to the temporal evolution of the region irradiated with continuous beam and bias are minimal. A possible explanation for the observed difference in the recovery of the CCE has been proposed: During normal operation of the detector when the bias is applied, the electric field is established between the electrodes. Without polarization effects, this electric field is homogeneous in the full volume of the device (planar geometry). When the shallow beam impinges in the top electrode, the atoms present in the lattice are ionized by ions and this process takes place close to the surface of the crystal. Under the influence of the applied electric field, the generated free carriers start to move but, depending on the polarity, only one type of free carrier will travel through the full detector being the main responsible for the induced signal. During their motion, some of these carriers will be captured, increasing the build-up of space charge. This generates an opposite electric field, which affects the biased electric field, according to the superposition principle, and the recombination rate increases leaving a deficit in the collected charge. In the experimental data, this corresponds to a continuous reduction in the detected signal over time when the bias and the beam are on during the first stage of the irradiation. 

Now the applied bias is switched off. Depending on whether the ion beam was also switched off or not, two different behaviors were observed:

**Beam On** If the bias is switched off but the beam continues irradiating the sample, new free carriers are generated. Now, in the unbiased detector, only the electric field induced by the accumulated space charge exists and, since it has an opposite direction to the biased electric field, the other type of free carriers starts to move through the detector. During this motion, recombination occurs with the captured carriers, decreasing the buildup space charge inside the diamond crystal and, in consequence, the polarization electric field. When the electric field due to bias is established again by switching the bias on, the conditions inside the detector are similar to the initial conditions because a significant reduction in the polarization electric field has happened due to a reduction in the accumulated space charge. For this reason, the recovery of the CCE is more efficient when the ion beam continues irradiating the detector after the bias has been shut down.
**Beam Off** If the bias and the beam are switched off simultaneously, no new free carriers are generated during these periods. The absence of new free carriers moving in the volume makes the recombination process much weaker, and the space charge build-up remains almost constant. When the bias is re-established again, the conditions in the detector are roughly similar (the space build-up charge is the same) and the recovery is less apparent. In addition, this explains why the CCE starts approximately at the same value as the one reached just when the bias was switched off.

**Figure 10:**
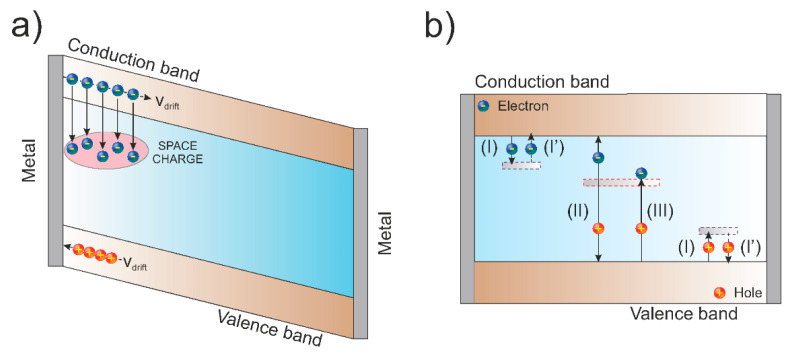
(**a**) Simplified image of the charge carriers transport and space charge accumulation under negative bias for a shallow ion beam; (**b**) diagram with the mechanisms of trapping (I), generation (II), recombination (III), and re-emission of charge carriers (I’).

Schematic diagrams showing the charge carrier transportation phenomenon (for negative polarity) [51] and generation/recombination mechanisms are summarized in Figure 10a,b.

These effects are more visible when the polarization is induced by holes (positive bias) as it was for our particular detector.

### 3.4. Alternating Bias

**Figure 11:**
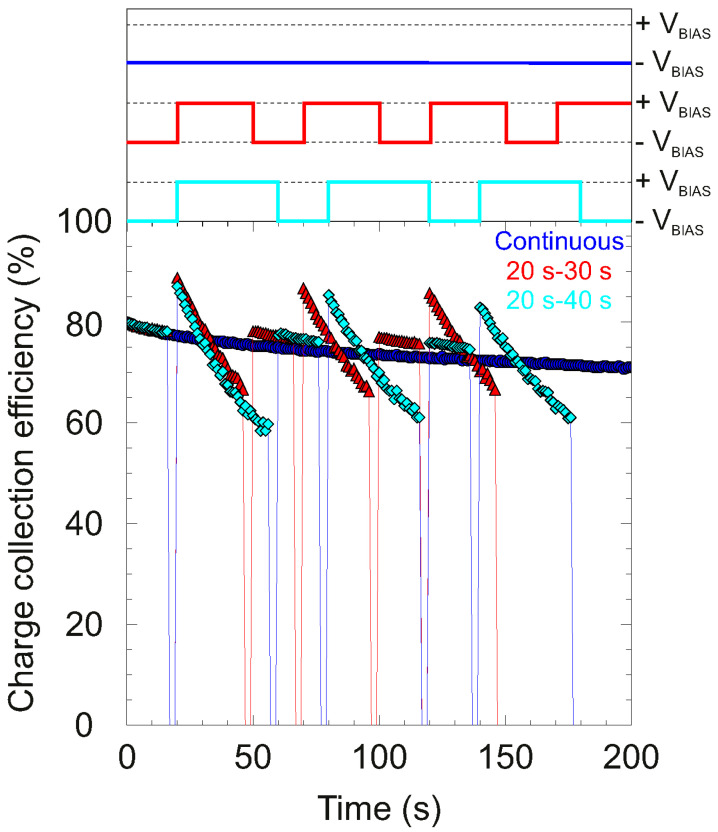
(**top**) Chronogram of the applied polarities; (**bottom**) temporal evolution of the CCE using alternating bias for different duty cycles. The temporal evolution under normal conditions (blue points) is also included for comparison.

An HV alternating unit was used to study depolarization effect through the use of periodic rectangular pulses changing the bias polarity during continuous irradiation. For this purpose, selective areas of the 65 μm detector were irradiated continuously with 4.0 MeV C^3+^ as a shallow probing ion beam. During the processing analysis, each pulse height histogram was recorded for 1 s. In these measurements, the applied bias polarities in the sample changed from positive to negative with the duty cycles, as shown in Table 1 or in the chronogram in Figure 11. Additionally, Figure 11 also summarizes the temporal evolution of CCE at different duty cycles of the alternating bias. All the measurements were performed at room temperature and without illumination. The current design of the alternating HV device started only from voltages with negative polarity, therefore, for comparison reasons, the measurement using continuous bias and continuous beam was only performed with negative polarity, as can be seen in Figure 11.

As can be seen from the figures described above, an overall continuous decrease in CCE was observed immediately after the irradiation started for the continuous case.

For the measurements in which the bias is alternated, the irradiation starts with the bias set to −12 V (E = −0.18 V/μm). The temporal evolution during the first 20 s is the same as in the continuous case, starting from around 80% of CCE. After the bias switches the polarity to positive, the CCE starts from a higher value (around ≈87%), which is compatible with the initial CCE when a positive bias is applied, as shown in [27].

This difference in the CCE value is due to the different probabilities of trapping electrons and holes. With positive polarity, it is clear that polarization becomes stronger than with negative bias, as can be seen in the abrupt drop in signal. After changing polarity again, no significant restoration of the signal for electrons was reached, starting the CCE value one small fraction above the last value obtained with negative bias. In contrast to the strong polarization induced by holes, a slow drop in the CCE value was observed after polarity was established for electrons. However, according to the results, when positive bias is applied again, a significant, but not complete, recovery of the CCE can be observed. In this recovery, the CCE always reaches a higher value with respect to the last value at the same polarity, which is an indicator that a significant portion of the accumulated positive space charge is removed during the cycle with negative polarity. In general, applying an alternating bias shows that the efficiency to remove the accumulated space charge is higher for holes than for electrons, unfortunately, strong polarization rapidly resumes when the bias is applied in the case of holes.

For this reason, an appropriate selection of duty cycles proves to be crucial for improving depolarization for optimal operation of a diamond detector.

### 3.5. Optical Excitation

**Figure 12:**
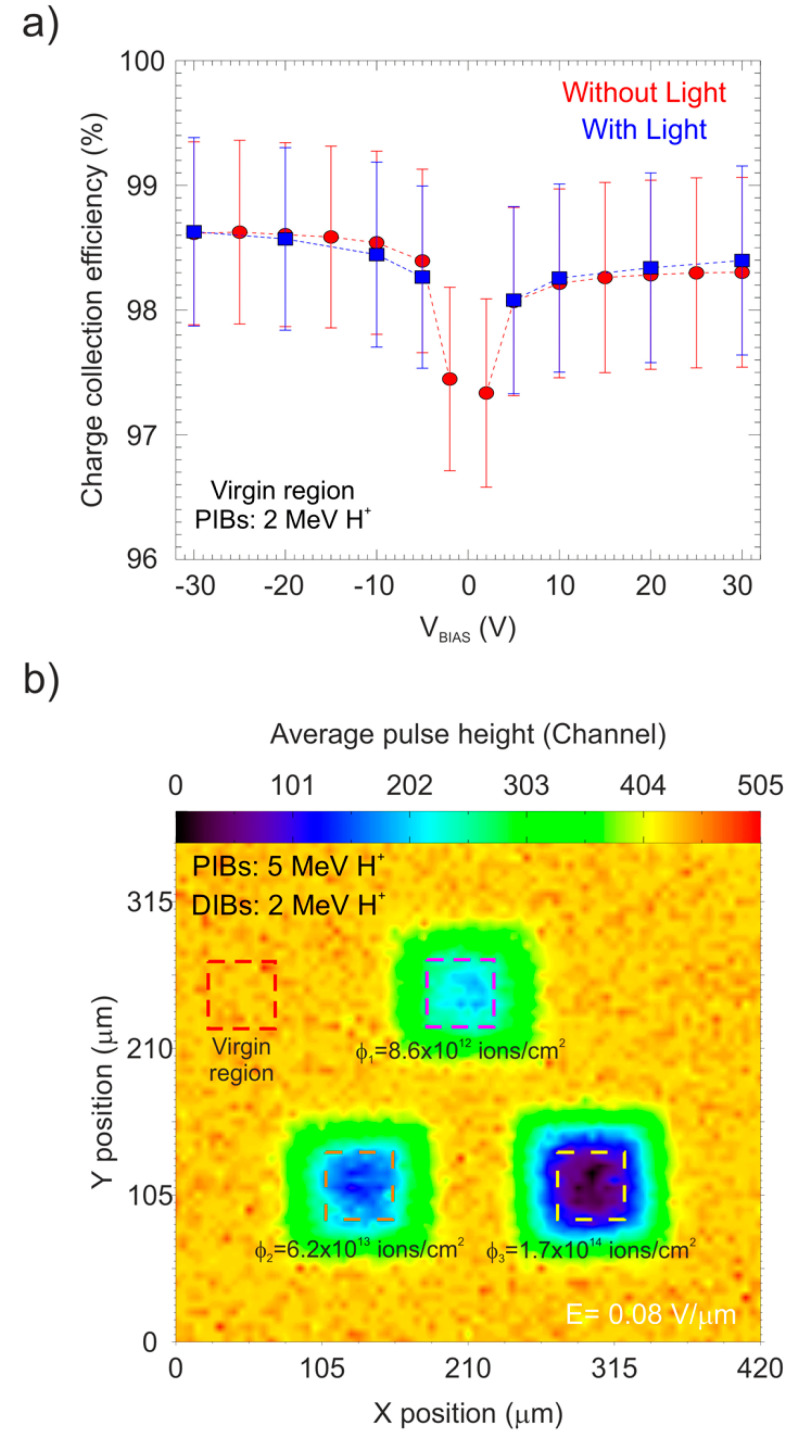
(**a**) CCE as a function of the applied bias for the pristine region of the 65 μm detector with (blue) and without (red) illumination; (**b**) IBIC map of the detector biased at +6 V (E = 0.09 V/μm) obtained using a 2 MeV proton beam showing the selected ROIs.

To study the influence of optical excitation on the temporal evolution of CCE during polarization, different ROIs were defined within the damaged regions and in one clean region of the pristine area of the 65 μm detector. As was mentioned in the Introduction, standard light does not affect the spectroscopic properties of a diamond detector, however, to confirm this, the CCE value of the virgin regions of the detector was studied as a function of the applied bias for both polarities with and without illumination. Figure 12a shows the relation between the CCE values for the detector biased from −30 V (E = −0.46 V/μm) to 30 V (E = 0.46 V/μm).

The error bars are defined as the full width at half maximum of each pulse height spectrum. From the figure, it is clear that the detector maintains a good spectroscopic performance, showing how the CCE rapidly reaches the saturation level (maximum CCE) for bias higher than ±2 V. In addition, the results illustrate that the applied bias polarity has little effect on the reported values of the CCE. No significant difference was observed in the CCE value during the application of light, which was a sign that white light did not have any effect on the spectroscopy behavior of the undamaged diamond detector, as would be expected due to the large band gap of the diamond crystal.

**Figure 13:**
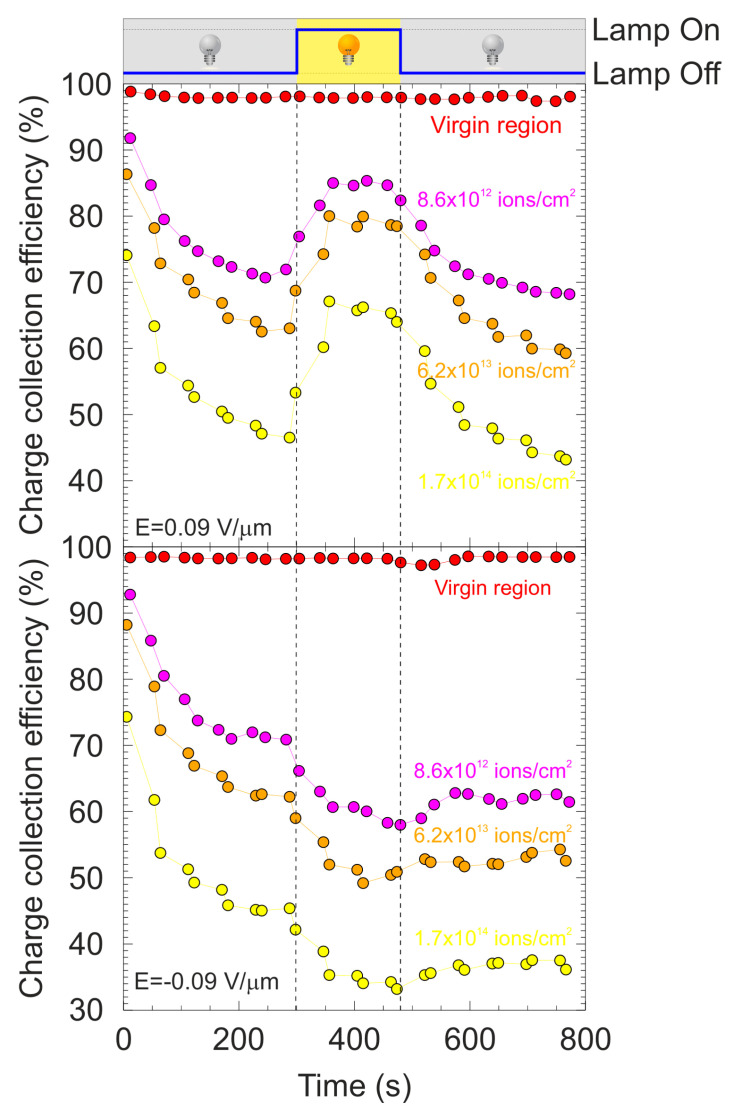
Temporal evolution of CCE for the different ROIs defined in the damaged regions for positive bias (**middle**) and for negative bias (**bottom**). The chronogram (**top**) indicates the applied illumination cycle.

To evaluate the same effect in the regions with different levels of damage, where the density of defects created in the crystal lattice increased the trapping efficiency, an analysis using the IBIC technique was carried out scanning an area of the detector of about 420 × 320 μm^2^ in size. This area contains the damaged regions probed by a 2 MeV proton beam with the detector biased at ±6 V (E = ±0.09 V/μm). In the resulting IBIC map shown in Figure 12b, the CCE is evaluated in the four ROIs which are marked with solid line squares with different colors. The effect of switching the light source on/off during the continuous irradiation on the evolution of CCE is presented in Figure 13 for positive and negative bias.

During the first 300 s from the beginning of the irradiation, continuous degradation of CCE was observed in all the regions except for the pristine region where the CCE remained constant. In Figure 13 above, it can be seen that polarization is clearly dependent on the amount of damage to the irradiated region, since the decrease is more remarkable in the region damaged with the highest fluence. This irradiation period was carried out in dark conditions. After the first 300 s, the light source was applied, continuously illuminating the full sample for 180 s. During this period, two different behaviors of CCE were observed depending on the applied polarity. For positive bias, a partial enhancement of CCE was observed for the damaged regions. The evolution of CCE during this cycle with illumination remained nearly flat for all the regions, which indicated that part of the accumulated space charge was removed. According to the experimental results, the recovery appears to be stronger for the most damaged region. On the one hand, no variation in CCE was observed in the pristine region, showing again that light does not have any effect in the undamaged zones of a diamond detector.

On the other hand, for the negative bias, an increase in the degradation of CCE due to illumination was reported. After this interval of time, the light was switched off again and the irradiation continued for another 300 s under dark conditions. For positive bias, the decrease in CCE rapidly resumed with an identical rate as that when the light was switched off for all the regions except, again, for the pristine region. However, for the negative bias, apparently, the drop did not persist, and it seemed that polarization was interrupted. This could be observed by the fact that no more degradation of CCE was observed after the light was switched off. The complete illumination cycle is represented in the chronogram shown in the top portion of Figure 13.

The absence of polarization in the pristine region can be explained by the purity (electronic grade) of the analyzed detector. Without radiation damage, the number of active traps (intrinsic defects or impurities) to capture free carriers is not enough to induce a significant distortion in the internal electric field [19]. For the damaged regions, the results now confirm the evidence that light induces almost instantaneous de-trapping of the hole traps, but not the electron traps for this particular detector. A study in literature review showed that the capability of depolarization using short wavelength light (as ultraviolet light) in high resistivity semiconductors [52], but this was due to the fact that the electromagnetic radiation in this spectral region had enough energy to ionize and/or excite the medium, creating new electron/holes pairs [53].

White light can only affect de-trapping, promoting electrons by optical transitions from trapped levels close to valence or conduction bands in the diamond crystals which helps to free the charge carriers captured in traps. However, the reason is still unclear why the CCE remained constant for negative bias (signal mainly created by electrons) when the light was removed after the illumination, indicating that trapping was interrupted even under continuous irradiation. This could be explained with the filling of all the traps during the irradiation, so the space charge accumulated remains constant and an equilibrium between the polarization electric field and the applied electric field is reached.

At this stage of understanding, we conclude that light has some effect activating/deactivating the traps, but only in the new traps (defects) created in the damaged regions. Taking into consideration that space charge accumulation depends on trapping probability which is a function of the density of traps, the de-trapping time, and the transport properties of the free carriers, such as their mobilities and their lifetimes, it is clear that light affects the trapping/de-trapping released rates of these energy levels in different ways [54].

A more systematic analysis is required for understanding this behavior. It is possible to use an interlinked combination of monochromatic light (for example, using LEDs) and the transient current technique (TCT) as proposed in [55] to study the electric field profile and the level of distortion due to positive or negative accumulated charge. In addition, it is important to characterize the defect levels induced by irradiation in diamond.

## 4. Conclusions

In this study, the IBIC technique with an ion beam microprobe was successfully applied to study the ion beam-induced polarization phenomena in two different diamond detectors. In particular, the main aim was to provide extensive information related to different polarization quenching techniques applied to sc-CVD diamond samples. This set of techniques historically has been used for depolarization in high-resistivity semiconductors such as the CdTe. This compilation of approaches results in a definition of the optimal method to depolarize diamond detectors during working operation according to the experimental results achieved in this study.

Our experimental results indicate that turning off the bias, and then reapplying it during continuous irradiation can be used as a satisfactory method for recovering CCE to a prepolarized state when polarization is induced by hole drift. This mitigation of polarization is due to a complete or partial reduction in the accumulated space charge, which re-establishes the internal electric field and the transport conditions of the holes within the sample. In contrast, the recovery that was obtained for polarization induced by electrons is weaker. For the case where the ion beam is also interrupted, the analysis leads to the following conclusions: The recovery of the signal for holes is significantly smaller as compared with the case when the ion beam is irradiating the sample. Again, no significant effect appeared for electrons. As discussed, this is because no new electron/hole pairs are being created during the period of time where the beam is off. This inhibits the probability of recombination with the trapped free carriers, which do not allow a reduction in the accumulated space charge. For this reason, the polarization effect remains after the beam is applied again.

Heating of the detector, or keeping it continuously at an elevated temperature, is also a particularly promising method for reducing the adverse effect of polarization. The results obtained in this study showed that, for our particular detector, a reduction in the space charge could be achieved by heating the sample to temperatures above 90 °C.

This can be explained by the release of free carriers from the trap levels induced by thermal excitation, giving the surrounding temperature enough energy to the carrier to escape and continue the drift motion reducing the density of the accumulated space charge. It is important to highlight the fact that the removed space charge is independent of the operating temperature, at least for the temperatures explored in this study, which shows that the temporal evolution of CCE has the same profile for temperatures close to 100 °C and 200 °C. Future studies should aim to confirm results in a larger temperature range reaching temperatures up to 400 °C, however in that case, increased leakage current could degrade the detector response. Furthermore, study of the temporal evolution of TCT signals during polarization at different temperatures could provide additional information about the charge transport properties.

The application of alternating bias during continuous irradiation has revealed that no significant effect on reducing the polarization phenomena took place, showing no significant recovering for polarization induced by electrons and partial recovery for holes. This is due to the strongest effect of polarization induced by holes, which quickly resumes when the polarity is changed to positive.

However, appropriate matching of duty cycles lengths for particular working conditions may result in a satisfactory mitigation strategy.

Finally, it has also been shown that illumination with standard light can be used as a method to suppress the strength of polarization induced by hole traps in damaged regions. A widely accepted explanation for a decrease in accumulated space charge during illumination of a sample is that white light has enough energy to promote and release the captured holes from the trap levels. Unfortunately, we found the opposite behavior for electrons. The observed difference in the behavior of the two charge carriers can only be attributable to the change in the electric field profile within the detector after the illumination was applied. The suppression of polarization effects induced by electrons after illumination of the sample is still unclear and requires further investigation.

## Figures and Tables

**Table 1:** Experimental configuration for the alternating bias tests.

Test	V_BIAS_ (V)	T_−_ (s)	T_+_ (s)
A	±12	20	30
B	±30	20	40

T_−_ refers to the period where the applied bias has negative polarity. T_+_ refers to the period where the applied bias has positive polarity.

## Data Availability

Not applicable.
